# A histological study of atherosclerotic characteristics in age-related macular degeneration

**DOI:** 10.1016/j.heliyon.2022.e08973

**Published:** 2022-02-16

**Authors:** Jingchao Wang, Hongpeng Zhang, Jing Ji, Lixuan Wang, Wenxin Lv, Yuan He, Xuan Li, Guangyu Feng, Kinon Chen

**Affiliations:** a Key Laboratory of Biomechanics and Mechanobiology, Ministry of Education, Beijing Advanced Innovation Center for Biomedical Engineering, School of Biological Science and Medical Engineering, Beihang University, Beijing, 100083, China; b Department of Vascular Surgery, Chinese People's Liberation Army General Hospital, Beijing, 100853, China

**Keywords:** Histology, Staining, Atherosclerotic vascular disease, Atherosclerosis, Blood vessel

## Abstract

This study investigated the pathogenesis of age-related macular degeneration (AMD) using histological methods that are commonly used for atherosclerotic vascular disease (ASVD). 1 normal, 3 early dry AMD, and 1 late dry AMD eyes were obtained from the Lions Eye Bank of Oregon and systematically dissected. They were stained with hematoxylin and eosin, Oil red O, Masson, Elastica van Gieson, Alizarin red, and Prussian blue. Additionally, the normal and late dry AMD eyes were immunostained for a-smooth muscle actin, CD45, and CD68 with Nile red and DAPI. Correlations were found between severity of AMD and lipid accumulation in the deep sclera (+), numbers of drusen between the Bruch's membrane and retinal pigment epithelium (RPE) (+), amount of collagen in the deep sclera (+), and amount of elastin in the deep sclera (-) (P < 0.1). Geographic atrophy, RPE detachment, and abnormal capillary shape and distribution in the choriocapillaris were observed in the fovea of late AMD. There were no stenosis, plaque, hemorrhage, and calcification. Additionally, late AMD tended to have higher smooth muscle thicknesses of the choroidal vascular walls, lower numbers of T lymphocytes in the choroid, and higher numbers of macrophages near the RPE and in the choroid relative to normal (P < 0.1). Macrophages-derived foam cells were detected near the Bruch's membrane in late AMD. Therefore, the present study showed many histological characteristics of ASVD in AMD, which suggests an association between them; however, there were also some histological characteristics of ASVD that were not found in AMD, which indicates that there exist pathogenic differences between them. The results generally support the vascular model of AMD, but some details still need clarification.

## 1 Introduction

Age-related macular degeneration (AMD) is one of the leading causes of visual impairment and blindness especially among older people worldwide [1,2]. As a consequence of population ageing, it is estimated that the number of people with AMD will be increased from 196 million in 2020 to 288 million in 2040 globally [3]. Unfortunately, our understanding of AMD pathogenesis is still far from complete due to the complexity of the disease.

It has been speculated that AMD is related to atherosclerosis (also known as atherosclerotic vascular disease or ASVD). The theory can be traced back to 1905, when Possek proposed that atherosclerotic changes of choroidal blood flow is a possible cause of AMD [4]. Later studies found that AMD and ASVD are indeed associated with each other epidemiologically [5, 6, 7, 8, 9, 10]. In addition, it was reported that the mechanisms of AMD and ASVD (cellular oxidative stress, endothelial dysfunction, inflammatory response, and lipoprotein retention), the histological and biochemical characteristics of their deposits (i.e., drusen and plaque), and their genetic factors share many similarities [11, 12, 13, 14, 15]. Furthermore, we recently discovered that AMD retina, choroid, and sclera exhibit abnormal mechanical properties that are similar to those seen in the atherosclerotic process [16, 17, 18]. However, the relationship between AMD and ASVD is still unclear.

ASVD has been commonly defined and classified by the characteristics of lipid accumulation, inflammatory response, plaque development, smooth-muscle proliferation, fibroproliferation, elastic-fiber changes, tissue thickening, foam cell formation, calcification, and hemorrhage with histology [19, 20, 21]. Although AMD has been studied with histology, it has never been comprehensively performed to examine the characteristics of ASVD in AMD eyes, which may help to better understand their relationship.

The aim of this study is to investigate the pathogenesis of AMD using histological methods that are commonly used for ASVD.

## 2 Materials and methods

### 2.1 Tissues

The study was approved by the Biological Science and Medical Engineering Ethics Committee of the Beihang University. Three early dry AMD eyes (3 males, 89 ± 2 years old), one late dry AMD eye (1 female, 94 years old), and one normal eye (1 male, 91 years old) were obtained from the Lions Eye Bank of Oregon (Portland, OR). All donors were Caucasians. An eye-bank staff identified the AMD donors with a known history of AMD in the death reports, which contained the information of the severity and type of AMD diagnosed by ophthalmologists. The staff also identified the normal donor without a known history of AMD and with age that matched the AMD donors' as much as possible. The classification of these eyes for AMD was later confirmed in our histology with the presence of drusen and/or geographic atrophy of the retinal pigment epithelium (RPE) according to the literature [22]. According to the death reports, the causes of death were anoxic brain injury (1 normal), middle cerebral artery ischemic stroke (1 early AMD), advanced Alzheimer's disease (1 early AMD), and cardiac arrest (1 early AMD and 1 late AMD). In addition, each donor had at least two ASVD risk factors, including depression (1 normal and 1 late AMD), recurrent urinary tract infections (1 normal and 1 early AMD), chronic obstructive pulmonary disease (1 early AMD), chronic kidney disease (1 early AMD and 1 late AMD), hypertension (1 early AMD and 1 late AMD), anxiety (1 late AMD), smoking (1 early AMD and 1 late AMD), and alcohol consumption (1 late AMD). Exclusion criteria were any other remarkable ocular history and any infectious disease such as hepatitis, HIV, syphilis, HTLV, and COVID-19. The eyes were preserved within 8 h of death. They were fixed in Davidson's fixative (Electron Microscopy Sciences, Hatfield, PA) for 36 h, rinsed with distilled water for 1 min, and stored in vials with 10% neutral buffered formalin (Medical Chemical Corporation, Torrance, CA). They were then shipped to our facility in foam boxes with dry ice.

In addition, some fresh atherosclerotic and normal blood vessel tissues of human coronary artery intima was obtained from the Chinese People's Liberation Army General Hospital (Beijing, China). The tissues were fixed in 4% formaldehyde solution and transported to our facility.

### 2.2 Sectioning

**Figure 1:**
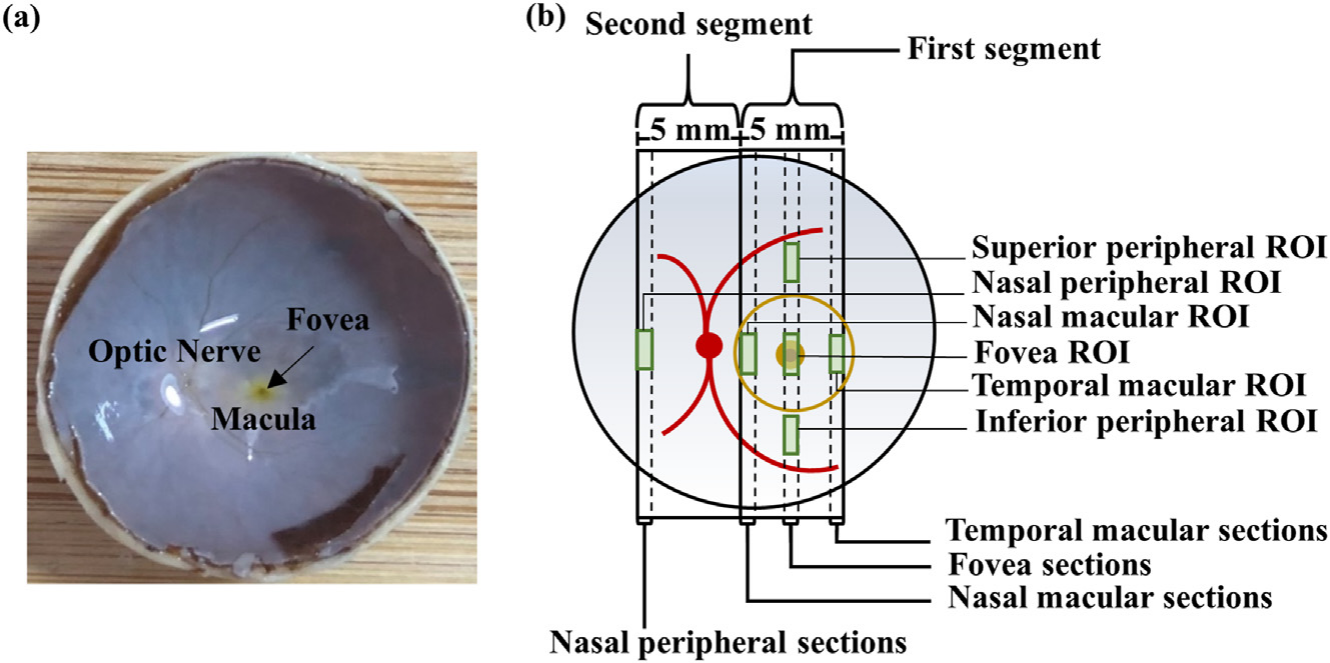
Eye dissection. After removing the anterior tissues and the vitreous, the posterior landmarks became visible (a). Two tissue segments were dissected out based on the landmarks, and tissue sections were collected from four different areas (b).

The eyes were removed from the vials and washed in a series of graded sucrose solutions. The anterior tissues and the vitreous were removed by dissection, and the remaining eye cups were trimmed to gain access to the posterior tissues (Figure 1a). Two 5-mm-wide segments were cut out of the posterior tissues superior-inferiorly, with the first segment that was centered at the fovea and the second segment that was immediately nasal to the first segment containing the optic nerve head (Figure 1b). The segments were dried with filter paper, embedded in OCT compound (Servicebio, Wuhan, China), frozen, and sectioned every 10 μm nasal-temporally in a cryostat at -20 °C. Sections containing the nasal peripheral, nasal macular, fovea, and temporal macular tissues were particularly selected for further processing (Figure 1b). In addition, the blood vessel tissues were sectioned using the same procedure for further processing.

### 2.3 Staining

The slides containing the selected sections were reheated and dried. Some slides were stained with various dye kits (Servicebio) to examine different pathological characteristics of the normal, early AMD, and late AMD eyes (Table 1). For hematoxylin and eosin (H&E) staining, the slides were stained with hematoxylin solution for 5 min, treated with hematoxylin differentiation solution, treated with hematoxylin Scott's tap bluing, immersed in 85% ethanol for 5 min, and rinsed with tap water between each step. They were immersed in 95% ethanol and eosin dye for 5 min each, dehydrated with 3 changes of 100% ethanol and 2 changes of xylene for 5 min each, and sealed with neutral gum.

**Table 1:** List of histological methods used in this study.

Stains/Immunostains	Effects
H&E	Cytoplasm (red); Nuclei (blue)
Oil red O	Lipid droplets (red); Nuclei (blue)
Masson	Collagen fibers (blue); Muscle fibers (red)
EVG	Elastin fibers (purple/black); Collagen fibers (red)
Alizarin red	Calcium salt deposit (dark red)
Prussian blue	Hemorrhage, Fe^3+^ (blue); Nuclei (red)
a-SMA; Nile red; DAPI	Smooth muscle marker (green); Lipid (red); Nuclei (blue)
CD45; Nile red; DAPI	T Lymphocytes marker (green); Lipid (red); Nuclei (blue)
CD68; Nile red; DAPI	Macrophages marker (green); Lipid (red); Nuclei (blue)

For Oil red O staining, the slides were stained with oil red O solution for 10 min in the dark, rinsed in 2 changes of 60% isopropanol for 3 s and 5 s respectively, and immersed in 2 changes of pure water for 10 s each. They were immersed in hematoxylin for 5 min, rinsed in 3 changes of pure water for 5 s, 10 s, and 30 s respectively, treated with differentiation solution (60% ethanol as solvent) for 8 s, rinsed in 2 changes of distilled water for 10 s each, treated with Scott's tap bluing for 1 s, and dipped in 2 changes of tap water for 5 s and 10 s respectively. The slides were sealed with glycerin gelatin.

For Masson staining, the slides were soaked in Mason A overnight, stained with a 1:1 mixture of Masson B and Masson C for 1 min, differentiated with 1% hydrochloric acid alcohol, soaked in Masson D for 6 min, soaked in Mason E for 1 min, and rinsed with tap water between each step. They were soaked in Mason F for 30 s, rinsed with 1% glacial acetic acid, dehydrated with 2 changes of anhydrous ethanol, immersed in 100% ethanol and xylene for 5 min each, and sealed with neutral gum.

For Elastica van Gieson (EVG) staining, the slides were stained with a 5:2:2 mixture of EVG A, EVG B, and EVG C for 5 min, rinsed with tap water, and treated with double diluted EVG B and tap water repeatedly until no change in color was observed. They were stained with a 1:9 mixture of EVG D and EVG E for 3 min, washed with tap water, dehydrated with 3 changes of anhydrous ethanol, immersed in 2 changes of xylene for 20 s and 5 min respectively, and sealed with neutral gum.

For Alizarin red staining, the slides were stained with alizarin red staining solution for 5 min, washed with tap water, and dried in an oven. They were dehydrated with xylene for 5 min and sealed with neutral balsam.

For Prussian blue staining, the slides were stained with a 1:1 mixture of Prussian blue staining solution A and solution B for 1 h, washed twice with distilled water, stained with Prussian blue staining solution C for 3 min, and washed with running water. They were dehydrated with 3 changes of 100% ethanol and 2 changes of xylene for 5 min each and sealed with neutral balsam.

### 2.4 Immunostaining

Some slides were immunostained with various reagents (Servicebio) to examine different pathological characteristics of the normal and late AMD eyes (Table 1). One set of slides was immunostained with primary antibodies against a-smooth muscle actin (a-SMA) (mouse, 1:500) in combination with secondary antibody conjugated with Alexa Fluor 488 (goat anti-mouse, 1:400), one set of slides was immunostained with primary antibodies against CD45 (rabbit, 1:500) in combination with secondary antibody conjugated with Alexa Fluor 488 (goat anti-rabbit, 1:400), and one set of slides was immunostained with primary antibodies against CD68 (rabbit, 1:200) in combination with secondary antibody conjugated with Alexa Fluor 488 (goat anti-rabbit, 1:400). The three sets of slides were blocked with 3% bovine serum albumin for 30 min, incubated with primary antibody overnight at 4 °C, washed three times with phosphate buffered solution (PBS) for 5 min each, incubated with secondary antibody for 50 min at room temperature, and washed three times with PBS again. In addition, they were incubated with Nile red for 10 min, incubated with DAPI for 10 min, sealed with antifade mounting medium, and washed three times with PBS between each step.

### 2.5 Imaging

All the slides for each histological method were stained or immnostained at the same time. Then, they were imaged using a Pannoramic MIDI digital slide scanner (3DHistech, Budapest, Hungary; Plan-Apochromat 20x, 0.274 μm/pixel) on the same day.

The LFB-stained sections were examined under a light microscope (Axioplan 2, Carl Zeiss, Jena, Germany) (5x, NA 0.15) while the NF/Tub/MBP-stained sections and the NeuN/Fluoro Nisslstained sections were examined under a fluorescence microscope (AxioObserver Z1 inverted confocal, Carl Zeiss) (myelinated axons: Plan-Apochromat 63x, NA 1.4, dorsal horn cells: Plan-Apochromat 20x, NA 0.8, ventral horn cells: Plan-Apochromat 10x, NA 0.45).

The imaging settings were fixed for each histological method. The images were viewed using Case Viewer software (3DHistech).

### 2.6 Data analysis

Images of the stained and immunostained blood vessel tissues were used as controls to ensure proper staining. Images of the stained and immunostained ocular tissues were processed for analysis. Specifically, the H&E-, Alizarin red-, and Prussian blue-stained ocular tissues were thoroughly examined to detect stenosis and plaque, calcification, and hemorrhage, respectively. The a-SMA/Nile red/DAPI and CD68/Nile red/DAPI-stained ocular tissues were thoroughly examined to detect foam cells.

In addition, the ratios of lipid, collagen, and elastin were calculated in the Oil red O-, Masson-, and EVG-stained deep scleral tissues, respectively. The numbers of hard, compound, and soft drusen were counted in the Oil red O-stained ocular tissue between the Bruch's membrane (BrM) and RPE according to the literature [23, 24, 25]. The smooth muscle thicknesses of the vascular walls were measured in the a-SMA/Nile red/DAPI-stained choroidal tissue (middle- and large-sized vessels only). The numbers of T lymphocytes and macrophages were respectively counted in the CD45/Nile red/DAPI- and CD68/Nile red/DAPI-stained tissues near the RPE and in the choroid. These analyses were performed in six regions of interest (ROIs) particularly: the nasal peripheral, nasal macular, fovea, and temporal macular ROIs were centered along the nasal-temporal axis of the fovea in the corresponding tissue sections, and the superior peripheral and inferior peripheral ROIs were 3 mm away from the fovea in the fovea section (Figure 1b). For the ratio analyses, boxes were placed adjacent to the inner side of the sclera (680 μm × 1260 μm for lipid, 150 μm × 500 μm for collagen, 340 μm × 1260 μm for elastin) in the ROIs, and the number of stained pixels was measured in the boxes using MATLAB (MathWorks, Natick, MA). For the other analyses, lines were drawn along the corresponding tissue layers (1000 μm for drusen, 1300 μm for smooth muscle thickness, T lymphocytes, and macrophages), and manual measurement was performed along the lines in Case Viewer by two independent observers who were blinded to the study design.

The relationships between disease severity (0 – normal, 1 – early AMD, 2 – late AMD) and staining outcomes were assessed using Spearman's rank correlation coefficient. In addition, immunostaining outcomes were divided into three groups (1, 0, -1 – above, within, and below the range of values of the normal eye among the six ROIs), and the relationships between normal and diseased (0 – normal, 1 – late AMD) immunostaining outcomes were assessed using a two-sided Fisher's exact test. Statistical analysis was performed using SPSS (IBM, Chicago, IL).

## 3 Results

### 3.1 Controls

**Figure 2:**
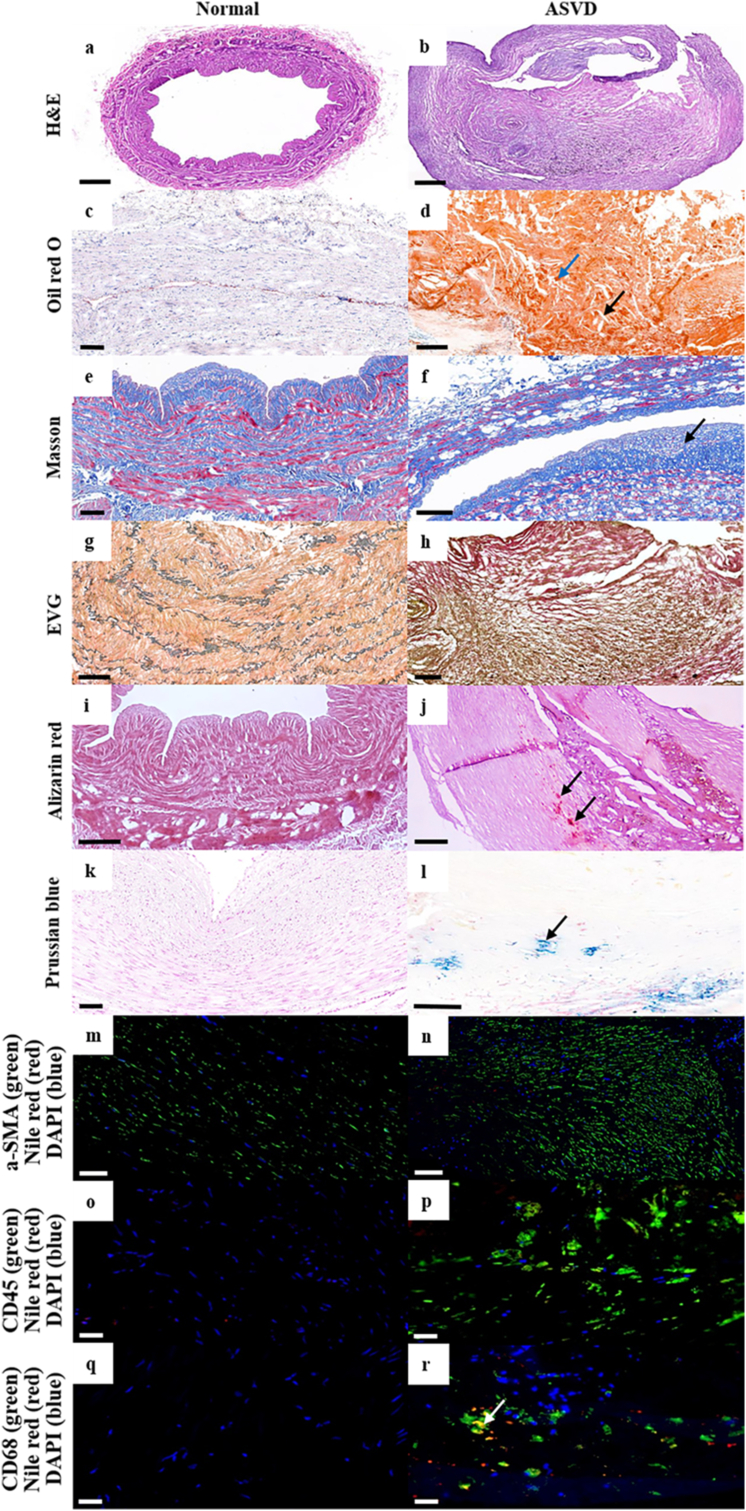
Representative images of normal and atherosclerotic blood vessel tissues of human coronary artery intima with different stains and immunostains. In atherosclerotic tissue, intimal thickening and plaque rupture were present in atherosclerotic lesions (b, scale bar = 400 μm) (b). Large amount of lipid droplets (blue arrow) and cholesterol crystals (black arrow) accumulated especially in plaque (d, scale bar = 200 μm). Fibroproliferation and fibrous cap (black arrow) (f, scale bar = 200 μm), elastin reduction (h, scale bar = 200 μm), calcium salt deposition (black arrows) (j, scale bar = 300 μm), and hemorrhage (black arrow) (l, scale bar = 100 μm) also clearly appeared in atherosclerotic tissue. Additionally, smooth muscle cells (n, scale bar = 100 μm), T lymphocytes (p, scale bar = 40 μm), and macrophages (r, scale bar = 40 μm) visibly increased in atherosclerotic tissue. Furthermore, macrophage-derived foam cells (white arrow) were detected by triple staining of CD68, Nile red, and DAPI (r). These characteristics were not seen in normal tissue (a, scale bar = 500 μm; c, scale bar = 100 μm; e, scale bar = 100 μm; g, scale bar = 200 μm; i, scale bar = 200 μm; k, scale bar = 100 μm; m, scale bar = 100 μm; o, scale bar = 40 μm; q, scale bar = 40 μm).

Distinct pathological characteristics were observed in atherosclerotic intima compared to normal intima with different stains and immunostains (Figure 2). Specifically, stenosis and intimal thickening, lipid accumulation, fibroproliferation, elastic-fiber reduction, calcification, hemorrhage, smooth muscle hyperplasia, T-lymphocyte infiltration, macrophage infiltration and macrophage-derived foam cell formation were observed with H&E, Oil red O, Masson, EVG, Alizarin red, Prussian blue, a-SMA/Nile red/DAPI, CD45/Nile red/DAPI and CD68/Nile red/DAPI (immuno)stains, respectively.

### 3.2 Lipid accumulation

**Figure 3:**
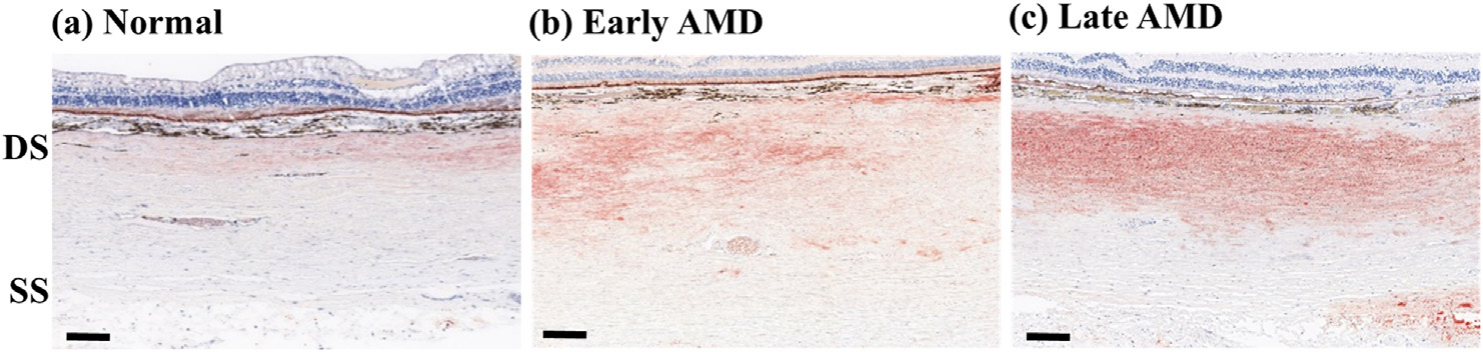
Representative images of Oil red O-stained ocular tissue in deep sclera. Lipids mostly accumulated in the deep sclera of the posterior eyes. The amount of lipid accumulation was visibly different between normal (a), early AMD (b), and late AMD (c). Furthermore, there appeared to be a gradual increase in the amount of lipid accumulation with distance from the choroid. DS, deep sclera; SS, superficial sclera. Scale bars = 200 μm.

**Figure 4:**
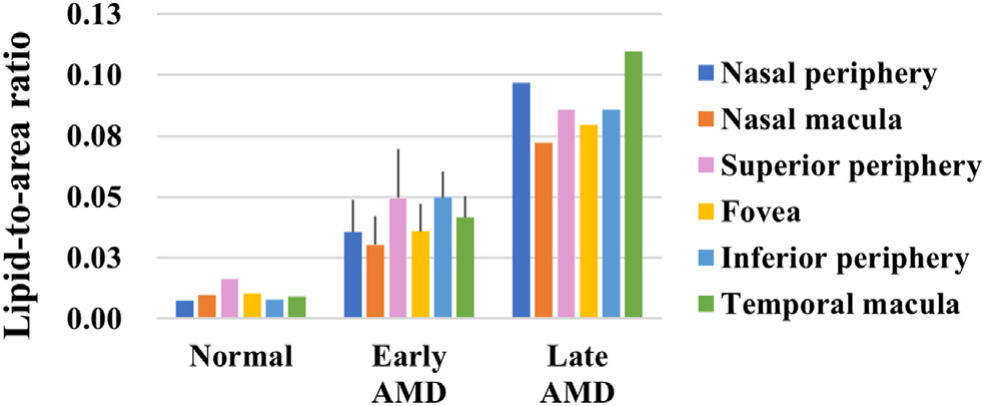
Ratio of lipid accumulation versus severity of AMD in deep sclera.

Positive correlations were found between the severity of AMD and the lipid accumulation in the deep sclera (p < 0.1, r_s_ = 0.924, 0.910, 0.868, 0.934, 0.963, 0.950 in the nasal peripheral, nasal macular, superior peripheral, fovea, inferior peripheral, and temporal macular ROIs) (Figure 3). Specifically, the ratios of lipid were 39.5–367.1% and 418.2–1213.3% respectively higher in late AMD than in early AMD and normal in the different ROIs, and they were 61.8–606.5% higher in early AMD than in normal (Figure 4).

### 3.3 Drusen

**Figure 5:**
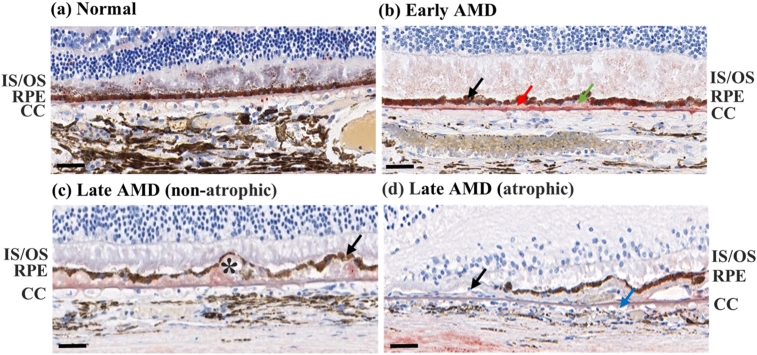
Representative images of Oil red O-stained ocular tissue between BrM and RPE. Drusen could hardly be seen in normal eye, and the RPE was intact (a). Hard drusen (red arrow), compound drusen (green arrow), and mild RPE disruption (black arrow) were seen in early AMD eyes (b). In addition to hard and compound drusen, empty spaces (asterisk), which were most likely soft drusen that had lost its lipid content during the fixation and dehydration procedures as previously reported, were detected only in late AMD eye (c) [57, 58]. Moreover, severe RPE disruption was seen in non-atrophic regions of late AMD (black arrow) (c). Geographic atrophy, RPE detachment (black arrow), and abnormal capillary shape (non-circular; blue arrow) and distribution (uneven) in the choriocapillaris were seen in the fovea of late AMD (d). IS/OS, inner and outer segments of photoreceptors; CC, choriocapillaris. Scale bars = 50 μm.

**Figure 6:**
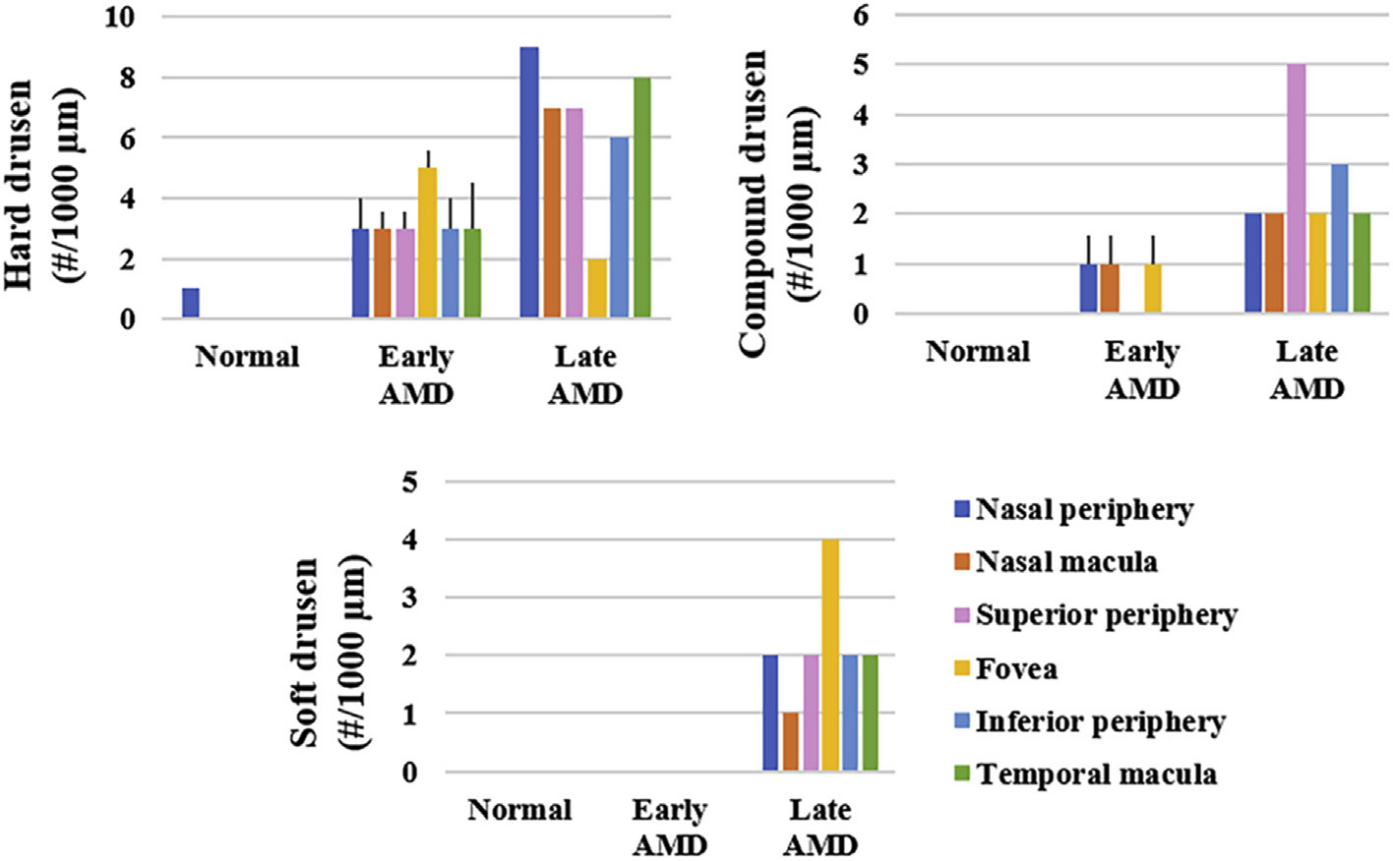
Numbers of drusen versus severity of AMD between BrM and RPE.

Positive correlations were found between the severity of AMD and the numbers of drusen between the BrM and RPE (p < 0.1, hard drusen: r_s_ = 0.801, 0.982, 0.986, 0.949, 0.886 in the nasal peripheral, nasal macular, superior peripheral, inferior peripheral, and temporal macular ROIs, compound drusen: r_s_ = 0.845, 0.845, 0.845, 0.791, 0.791, 0.791 in the nasal peripheral, nasal macular, superior peripheral, fovea, inferior peripheral, and temporal macular ROIs, soft drusen: r_s_ = 0.791, 0.791, 0.791, 0.791, 0.791, 0.791 in the nasal peripheral, nasal macular, superior peripheral, fovea, inferior peripheral, and temporal macular ROIs) (Figure 5). Particularly, there was only small number of hard drusen in the normal eye, and soft drusen only appeared in the late AMD eye (Figure 6).

### 3.4 Collagen

**Figure 7:**
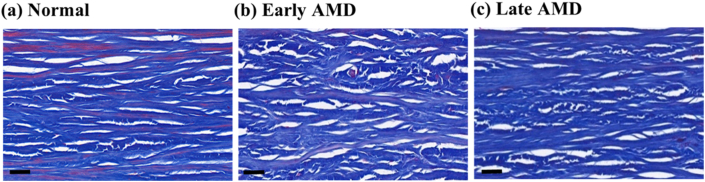
Representative images of Masson-stained ocular tissue in deep sclera. The amount of collagen was visibly different between normal (a), early AMD (b), and late AMD (c). Scale bars = 40 μm.

**Figure 8:**
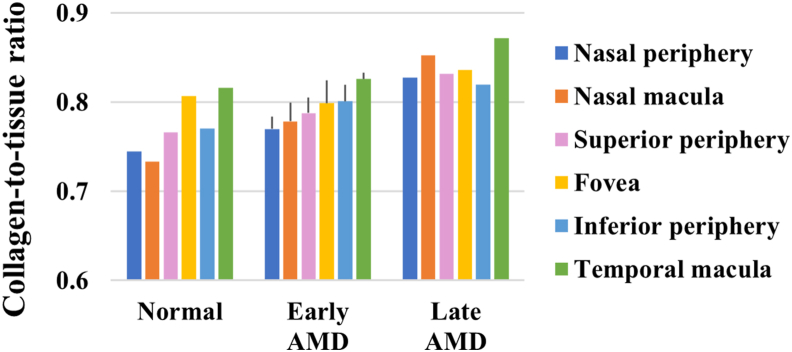
Ratio of collagen versus severity of AMD in deep sclera.

Positive correlations were found between the severity of AMD and the amount of collagen in the deep sclera (p < 0.1, r_s_ = 0.914, 0.928, 0.860, 0.846, 0.766 in the nasal peripheral, nasal macular, superior peripheral, inferior peripheral, and temporal macular ROIs) (Figure 7). Specifically, the collagen-to-tissue ratios were 0.2–13.0% and 6.3–16.2% respectively higher in late AMD than in early AMD and normal in the different ROIs, and they were 0.2–8.2% higher in early AMD than in normal in the non-fovea ROIs (Figure 8).

### 3.5 Elastin

**Figure 9:**
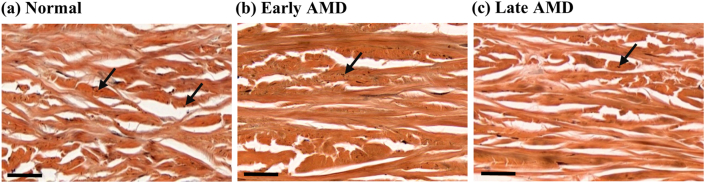
Representative images of EVG-stained ocular tissue in deep sclera. Elastin fibers (black arrows) were distributed in the space between collagen bundles, and the amount of elastin was visibly different between normal (a), early AMD (b), and late AMD (c). Scale bars = 40 μm.

**Figure 10:**
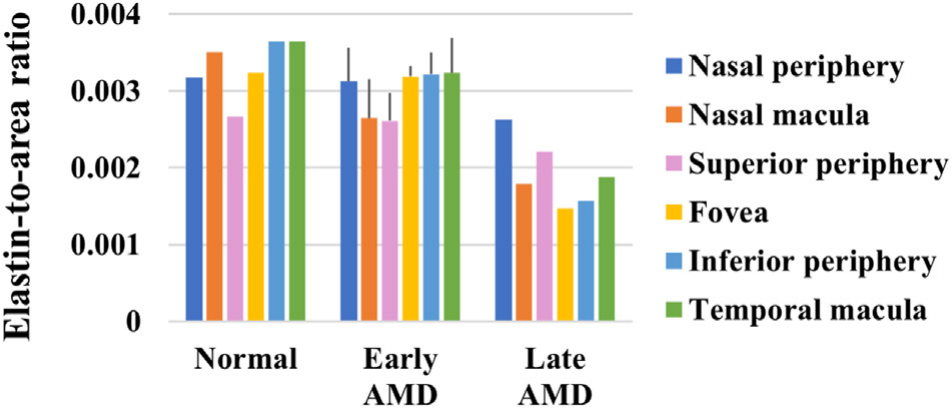
Ratio of elastin versus severity of AMD in deep sclera.

Negative correlations were found between the severity of AMD and the amount of elastin in the deep sclera (p < 0.1, r_s_ = -0.902, -0.8, -0.884, -0.834 in the nasal macular, fovea, inferior peripheral, and temporal macular ROIs) (Figure 9). Specifically, the elastin-to-area ratios were 17.3–55.7% and 48.4–57.0% respectively lower in late AMD than in early AMD and normal in the different ROIs, and they were 2.2–38.3% lower in early AMD than in normal in the nasal macular, inferior peripheral, and temporal macular ROIs (Figure 10).

### 3.6 Vascular walls

**Figure 11:**
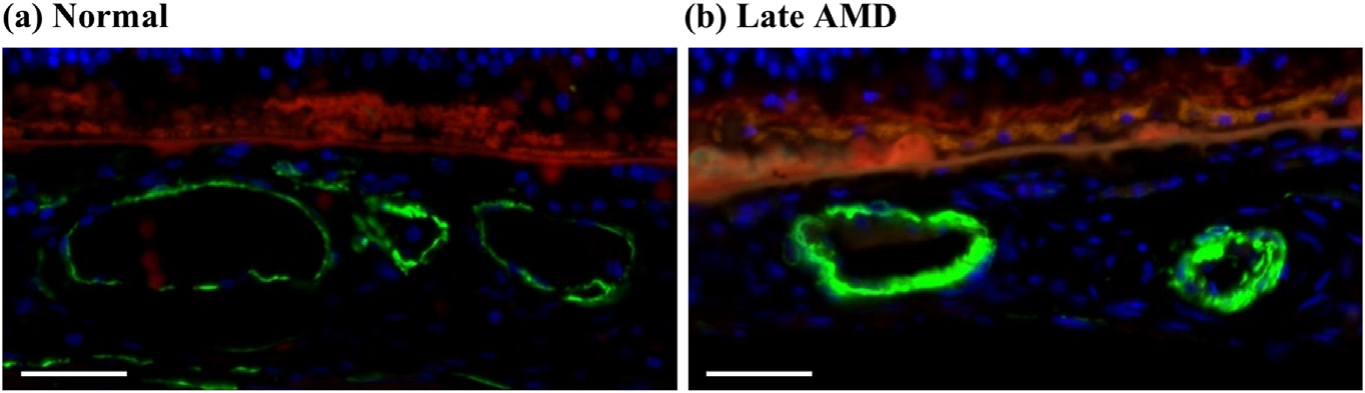
Representative images of vascular smooth muscle fibers in choroid. The vascular walls were visibly thinner in normal (a) compared to late AMD (b). Sections were labelled with a-SMA (green), Nile red (red) and DAPI (blue). Scale bars = 50 μm.

**Figure 12:**
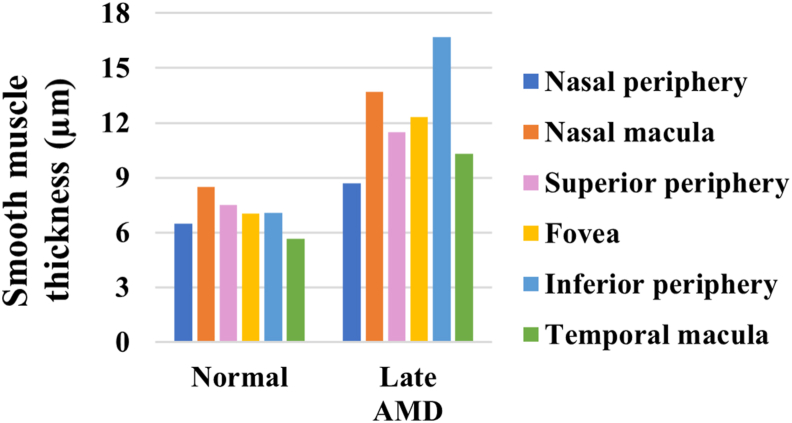
Smooth muscle thickness of vascular walls in normal and late AMD eyes in choroid.

The smooth muscle thicknesses of the vascular walls tended to be higher in late AMD relative to normal in the choroid (p < 0.1) (Figure 11). Specifically, the smooth muscle thicknesses were 33.8–136.5% higher in late AMD than in normal in the different ROIs (Figure 12).

### 3.7 T lymphocytes

**Figure 13:**
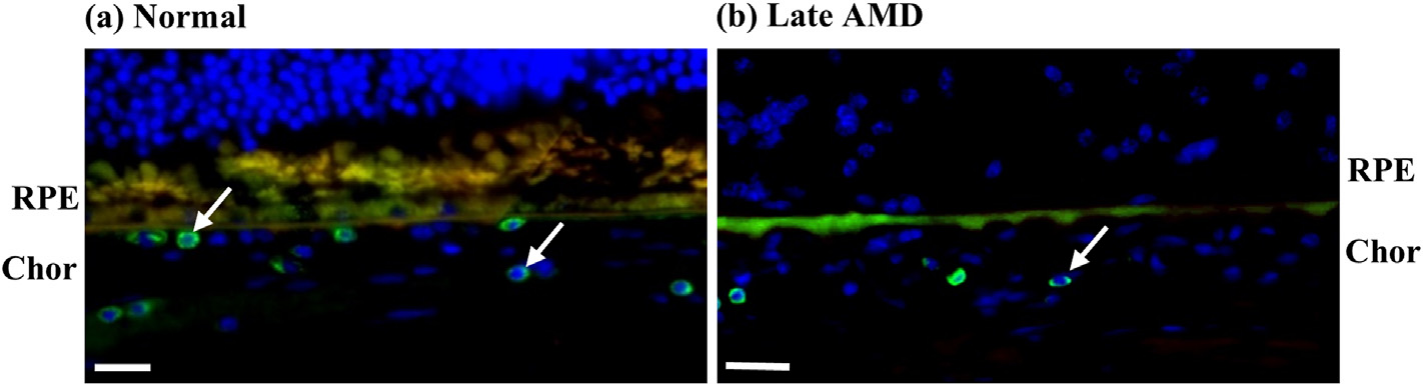
Representative images of T lymphocytes in choroid. There were visibly more T lymphocytes (white arrows) in normal (a) compared to late AMD (b). Sections were labelled with CD45 (green), Nile red (red) and DAPI (blue). Scale bars = 20 μm.

**Figure 14:**
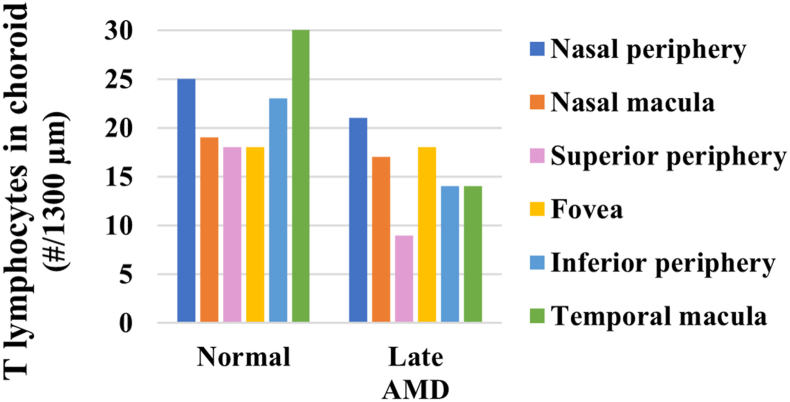
Number of T lymphocytes in normal and late AMD eyes in choroid.

The numbers of T lymphocytes tended to be lower in late AMD relative to normal in the choroid (p < 0.1) (Figure 13). Specifically, the T lymphocytes in the choroid were 16.0–53.3% lower in late AMD than in normal in the non-fovea ROIs (Figure 14). Also, no T lymphocytes were found near the RPE.

### 3.8 Macrophages

**Figure 15:**
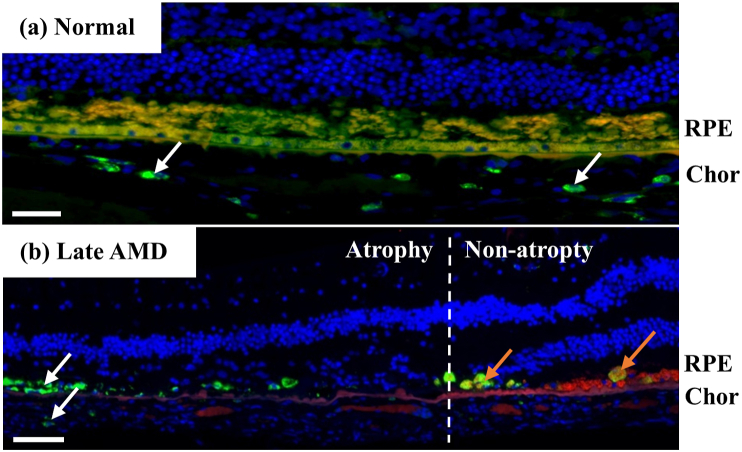
Representative images of macrophages near RPE and in choroid. In normal, macrophages were uniformly distributed in the choroid (white arrows), and they were rarely seen near the RPE (a). In late AMD, macrophages concentrated near the RPE (or where it used to be) and in the choroid in the atrophic region (white arrows) and non-atrophic region (orange arrows) (b). Sections were labelled with CD68 (green), Nile red (red) and DAPI (blue). Chor, choroid. Scale bars = 50 μm.

**Figure 16:**
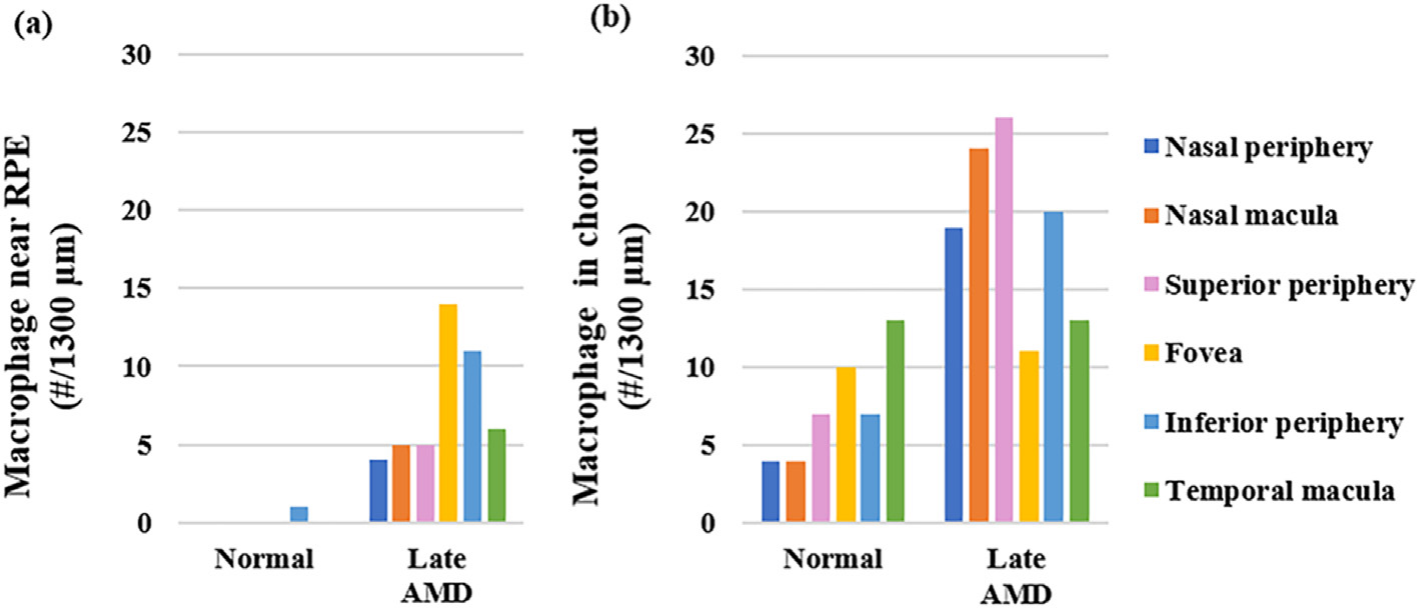
Numbers of macrophages in normal and late AMD eyes near RPE (a) and in choroid (b).

The numbers of macrophages tended to be higher in late AMD relative to normal near the RPE and in the choroid (p < 0.1) (Figure 15). Particularly, the macrophages were hardly seen near the RPE in normal, and they were 10.0–500.0% higher in late AMD than in normal in the non-fovea ROIs in the choroid (Figure 16).

### 3.9 Foam cells

**Figure 17:**
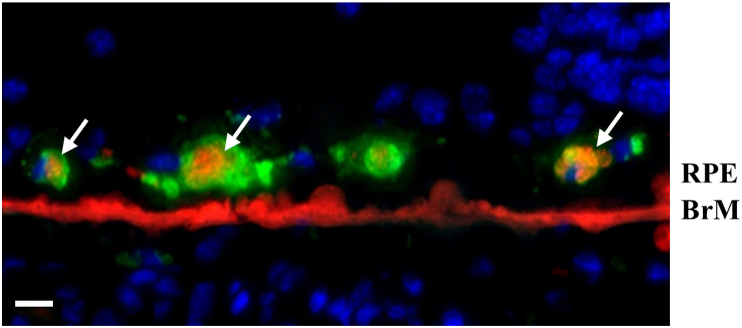
Macrophage-derived foam cells (white arrows) near BrM in late AMD. The cells were co-stained with CD68 (green), Nile red (red), and DAPI (blue). Scale bar = 20 μm.

A small number of macrophages-derived foam cells were found near the BrM where lipids accumulated in late AMD (Figure 17). The cell body size was typically between 20 and 40 μm in diameter. Smooth muscle-derived foam cells were not detected.

### 3.10 Other findings

Stenosis, plaque, hemorrhage, and calcification were not found in normal, early AMD, and late AMD.

## 4 Discussion

This study investigated the pathogenesis of AMD using histological methods that are commonly used for ASVD. The methods were validated with blood vessel tissues, and the results were consistent with previous studies [20,26,27]. Many characteristics of ASVD were found in the AMD eyes.

### 4.1 Lipids and drusen

Lipids are not frequently seen in the extracellular matrix of normal arterial and ocular tissues. However, with aging and ASVD, lipid deposits can increase dramatically in the arterial tissue [28]. In addition, it has been reported that the concentration and composition of lipids change with aging in the sclera; sphingomyelin and cholesterol esters increase in the perifibrous extracellular matrix of senile sclera that resembles the picture of lipid accumulation in the aorta with aging and ASVD to a certain extent [29]. The present data show that lipids accumulate also with AMD in the sclera that is close to the vascular choroid across different regions, which suggest that the phenomenon is more likely induced via systemic hyperlipidemia rather than just local lesions.

Drusen are considered the hallmark of AMD. Previous study of early AMD reported that hard drusen appeared in both macula and periphery, compound drusen appeared only in the periphery, and soft drusen appeared only in the macula [23]. Contrary to the previous findings, we found that both hard and compound drusen appeared in the macular and peripheral regions in early AMD, and soft drusen only appeared in late AMD. Moreover, it has been reported that confluence of soft drusen can cause RPE detachment and geographic atrophy, and alteration of the choriocapillaris may result in geographic atrophy, which coincide with our observations [30, 31, 32].

### 4.2 Elastin, collagen, and smooth muscles

Elastin and collagen are main components responsible for the mechanical behavior of the arterial wall and sclera [33,34]. With aging and ASVD, changes of collagen (generally increased) and elastin (increased, decreased, or relocated) can occur, which may affect the mechanical behavior [20,35]. The exact mechanical changes depend on other factors such as lipid accumulation and calcification as well [36, 37, 38, 39, 40]. We previously found that retinal elastin decreased and the mechanical properties of the retina, choroid, and sclera changed with late AMD [16, 17, 18]. The present data further show that the amount of elastin and collagen changes in the deep sclera with different severity of AMD.

Furthermore, it is known that smooth muscle cells proliferate as ASVD progresses, and the resulting thickening of the intima may lead to stenosis that reduces the blood flow [41,42]. Some studies have reported that the choroidal blood flow is reduced in AMD [43,44]. Here we present evidence that the choroidal vascular walls are indeed thickened from smooth muscle hyperplasia in AMD.

### 4.3 Immune cells

The pathogenesis of ASVD involves the deposition and modification of lipids within arterial walls and the chronic inflammatory response to these lipids [45]. The immune cells, such as T lymphocytes and macrophages, accelerate the progression of ASVD that plays a key role in the disease [45]. Previous studies reported that macrophages increased in the RPE and BrM of atrophic AMD, which were likely attracted by the accumulated lipids there [47,48]. However, previous study also reported that T lymphocytes actually decreased in the choroid of atrophic AMD [46]. The present data agree well with these findings in AMD.

Moreover, macrophages contribute to the pathogenesis of ASVD by internalizing native or modified lipoproteins or lipoprotein remnants that have invaded the vessel wall to form cholesterol-rich foam cells, which is known as a prominent feature of ASVD that promotes inflammation [26,49]. For the first time, this study discovered the existence of macrophage-derived foam cells in AMD. 7-Ketocholesterol, which is suspected of being involved in foam cell formation from macrophages in ASVD, was reported to be deposited in the choriocapillaris and BrM in primates, so it may be involved in the formation of these foam cells in AMD [50,51].

### 4.4 Hemodynamic theory of AMD

In 1997, Friedman proposed the hemodynamic (or vascular) model of AMD to theorize the role of ASVD in the pathogenesis of AMD [52, 53, 54, 55]. The model states that AMD is a vascular disorder, the hemodynamic sequela of atherosclerotic changes affecting the eye [52, 53, 54, 55]. AMD begins with age-related lipid infiltration of the sclera and BrM, which increases the stiffness of these tissues [52, 53, 54, 55]. As a result of the stiffening, resistance of choroidal vessels increases that impairs the RPE metabolism and transport [52, 53, 54, 55]. As the disease progresses, drusen and basal laminar deposits are formed increasingly between the inner surface of the BrM and the RPE that eventually causes geographic atrophy [52, 53, 54, 55]. Although some details of this model remain to be clarified, it is arguably the most popular theory for explaining the relationship of AMD and ASVD [17,18,56].

Here, we present histological evidences of lipid and drusen accumulation that are in agreement with the hemodynamic model of AMD. The elastin and collagen changes provide evidences that there are mechanical changes in AMD sclera, although we actually found it to be softened previously; nevertheless, resistance of choroidal vessel may still be disrupted as a result of the softening [17,18]. Furthermore, based on our results, alteration of the choriocapillaris and hyperplasia of choroidal vascular walls likely play important roles to affect the blood flow and pressure, and macrophages are likely involved in the pathogenesis of AMD that is similar to their involvement in the pathogenesis of ASVD.

### 4.5 Limitations and conclusions

The main limitations of this study are the small sample size and the lack of wet AMD samples. During the COVID-19 pandemic, collection of human eye samples was almost impossible. Conducting experiments also became difficult (e.g., the early AMD eyes were stored in formalin for too long to be properly immunostained during lockdown). Similar histological approach should be used in the future to further clarify the pathogenesis of AMD, especially the relationship between wet AMD and ASVD.

In conclusion, this study found many histological characteristics of ASVD in AMD, which suggests an association between these diseases.

## Declarations

### Author contribution statement

Jingchao Wang: Conceived and designed the experiments; Performed the experiments; Analyzed and interpreted the data; Wrote the paper.

Hongpeng Zhang: Conceived and designed the experiments; Analyzed and interpreted the data; Contributed reagents, materials, analysis tools or data; Wrote the paper.

Jing Ji: Conceived and designed the experiments; Analyzed and interpreted the data; Wrote the paper.

Lixuan Wang, Wenxin Lv, Yuan He, Xuan Li, Guangyu Feng: Performed the experiments; Analyzed and interpreted the data.

Kinon Chen: Conceived and designed the experiments; Performed the experiments; Analyzed and interpreted the data; Contributed reagents, materials, analysis tools or data; Wrote the paper.

### Funding statement

This work was supported by the Beijing Natural Science Foundation (project no. 7154215), the National Natural Science Foundation of China (project no. 81771347), and the National Natural Science Foundation of China (project no. 12172032).

### Data availability statement

Data will be made available on request.

### Declaration of interests statement

The authors declare no conflict of interest.

### Additional information

No additional information is available for this paper.
